# Lipidomic profiling reveals systemic serum lipid remodeling induced by dexamethasone in mice

**DOI:** 10.3389/fmolb.2025.1644637

**Published:** 2025-09-26

**Authors:** Aiping Zhang, Zhao Wang, Jianjian Ji

**Affiliations:** ^1^ Nanjing Jiangning District Center for Disease Control and Prevention, Nanjing, Jiangsu, China; ^2^ Jiangsu Provincial Research Institute of Chinese Medicine Schools, Jiangsu Key Laboratory of Children’s Health and Chinese Medicine, Institute of Pediatrics, Nanjing University of Chinese Medicine, Nanjing, China

**Keywords:** blood lipids, dexamethasone, Exactive Orbitrap-MS technology, lipidomic, lipid remodeling

## Abstract

**Background:**

Dexamethasone is a commonly used glucocorticoid medication in pediatrics. However, dexamethasone is associated with some side effects, such as dyslipidemia. This study aimed to explore the effects of dexamethasone on the serum lipidome.

**Methods:**

We utilized Exactive Orbitrap-MS technology to assess the effects of dexamethasone intervention on serum lipids in mice.

**Results:**

Unbiased Principal Component Analysis (PCA) revealed that dexamethasone intervention significantly induced changes in serum lipids in mice, and after a 7-day washout period (equivalent to 28 drug half-lives), changes of lipids in the serum were still existed compared with those in the control groups. After 4 days of dexamethasone injection, significant changes were observed, including 16 increased lipids, and 1 decreased lipid in the serum compared with those in the control groups. After a 7-day washout period, some of lipids in the serum were still changed, including 5 increased lipids, such as Acylcarnitines (CAR), ceramides (Cer), diacylglycerophosphates (DG), lysophosphatidylglycerol (LPG) and phosphatidylglycerol (PG), 1 decreased lipid, hexosylceramides (HexCer), indicating dexamethasone may result in long-term changes of lipids in the serum.

**Discussion:**

In conclusion, utilizing a lipidomics method, we provide the first complete proof that dexamethasone intervention generates extensive modification of the serum lipidome.

## 1 Introduction

Glucocorticoids like dexamethasone are widely used anti-inflammatory and immunosuppressive drugs (Prete and Bancos, 2021). Currently, an estimated 1%–3% of the global population takes glucocorticoid therapy (Prete and Bancos, 2021). The COVID-19 pandemic further spotlighted dexamethasone as a life-saving medication for extreme instances, dramatically lowering death in hospitalized patients (Li et al., 2023). However, long-term glucocorticoid usage is notorious for significant metabolic effects, including dyslipidemia and insulin resistance (Gasparini et al., 2019). Dexamethasone-induced dyslipidemia is characterized by elevations in total cholesterol, triglycerides, and low-density lipoprotein (LDL), often accompanied by modifications in high-density lipoprotein (HDL) (Rahimi et al., 2020; Yu et al., 2023). Clinical and animal investigations have consistently shown that glucocorticoid exposure perturbs lipid profiles—for example, chronic dexamethasone medication boosts blood LDL and triglycerides while altering HDL levels (Sholter and Armstrong, 2000). These lipid alterations are clinically significant: glucocorticoid-induced dyslipidemia is implicated in heightened cardiovascular risk (Sholter and Armstrong, 2000). Indeed, people on extended steroid therapy (e.g., for asthma or autoimmune disorders) frequently have a pro-atherogenic lipid profile with elevated LDL and triglycerides, which can accelerate atherosclerosis (Sholter and Armstrong, 2000).

Lipids are not only energy sources but also key regulators of cell communication, immune function, and tissue homeostasis (van Meer, 2005). Disordered lipid metabolism underpins numerous glucocorticoid side effects. Our recent lipidomics analysis demonstrated that dexamethasone affects pulmonary surfactant lipid composition, depleting phosphatidylcholine, phosphatidylglycerol, and phosphatidylethanolamine while raising ceramides and glycerides in bronchoalveolar fluid (Wang et al., 2024). This observation generated the question of whether systemic lipid networks are similarly affected (Wang et al., 2024). Notably, despite evidence of glucocorticoid effects on particular lipids and lipoproteins, it remains unclear if dexamethasone increases overall blood lipidome changes.

Here, we overcome this gap by applying high-resolution lipidomics to a mouse model of clinically relevant dexamethasone exposure. Using ultra-high-performance liquid chromatography connected to quadrupole-Orbitrap mass spectrometry (UHPLC-Q-Exactive Orbitrap-MS) in both positive and negative ion modes, we comprehensively examined serum lipids in dexamethasone-treated mice vs. controls. This approach enables us to capture unbiased, system-wide changes in lipid species. Our goal was to determine how short-term dexamethasone administration alters the serum lipidome, and to identify specific lipid pathways affected by glucocorticoids. The findings provide unique insights into the molecular links between dexamethasone prescription and dyslipidemia, highlighting possible biomarkers and mechanisms of glucocorticoid-induced metabolic deleterious effects. By describing these lipidomic changes, our study contributes to a better knowledge of glucocorticoid side effects and suggests options for limiting risks while retaining therapeutic advantages.

## 2 Method and materials

### 2.1 Animal model

**FIGURE 1:**
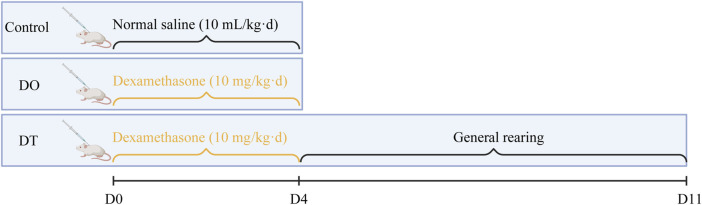
Experimental design figure.

The experimental protocol was approved by the Animal Ethics Committee of Nanjing University of Chinese Medicine (Approval No: 202204A002). SPF Balb/c female mice, aged 3–4 weeks and weighing 15.41 ± 0.89 g, were maintained under regulated settings (temperature: 24 °C–26 °C, relative humidity: 55%, and a 12-h light/dark cycle). Following a 3-day acclimatization period, mice were randomly randomized to three groups: DO (n = 16), DT (n = 16), and Control (n = 14). The DO and DT groups received daily subcutaneous injections of dexamethasone (Dex, 10 mg/kg), based on clinically identical dosage evaluations, for four consecutive days. The control group received an equivalent volume of saline. On day 5, the DO and control groups were killed for sample collection (Shen et al., 2014). The DT group underwent an additional 7 days under regular settings (equivalent to 28 half-lives of Dex) (Song et al., 2020) before euthanasia on day 11 (Figure 1). Clinical dexamethasone dosages vary greatly; for example, during acute exacerbations of childhood asthma, the dosage is 0.6 mg/(kg·d) (Paniagua et al., 2017), whereas in refractory chronic idiopathic thrombocytopenic purpura, the dosage may surpass 1 mg/(kg·d) (Chen et al., 1997). Thus, the chosen dosage attempted to resemble therapeutically relevant medicines applied in past trials.

### 2.2 Instruments and reagents

Vortex-Genie2 vortex mixer (Scientific Industries, Bohemia, NY, United States); Revco UXF ultra-low temperature freezer (Thermo Fisher Scientific, San Jose, CA, United States); CPA225D precision electronic balance (Sartorius, Göttingen, Germany); Allegra64R high-speed refrigerated centrifuge (Beckman, Pasadena, CA, United States); THZ-C thermostatic shaker (Taicang Qianle Experimental Equipment Co., Ltd., Taicang, Jiangsu, China); KQ-500B ultrasonic cleaning machine (Kunshan Ultrasonic Instrument Co., Ltd., Kunshan, Jiangsu, China); Savant SPD1010 vacuum concentrator (Thermo Fisher Scientific, San Jose, CA, United States); UHPLC-Q Exactive Orbitrap/MS and Xcalibur 2.1 SP1 software (Thermo Fisher Scientific, San Jose, CA, United States).

Lipopolysaccharide (Sigma, Santa Clara, CA, United States); Phosphate-buffered saline (PBS): sodium chloride (8.0 g), potassium chloride (0.2 g), disodium hydrogen phosphate (1.56 g), potassium dihydrogen phosphate (0.1 g) in 1,000 mL ultrapure water, autoclaved and cooled to room temperature; Methyl tert-butyl ether (MTBE, ROE, United States); Internal standards: lysoPE (17:1, LM171LPE-11) and PE (17:0/17:0, LM170PE-19, Avanti Polar Lipids, United States); Glacial acetic acid (99.5% purity, Nanjing Chemical Reagents Co., Ltd., China); Isopropanol, ammonium formate, ammonium acetate (99.8% purity, ROE, United States); Methanol and acetonitrile (99.8% purity, Merck, Germany) (Yang et al., 2019).

### 2.3 Sample collection

Mice were deprived overnight with free access to water prior to death. Under anesthesia, one eye was removed to acquire blood samples in 1.5 mL tubes. Samples were left at room temperature for 15 min, centrifuged at 3,500 rpm for 15 min, and serum was collected for lipidomic analysis.

### 2.4 Sample preparation

Serum samples (20 µL) were mixed with 225 µL ice-cold methanol containing internal standards (approximately 5 µg/mL lysoPE (17:1) for positive mode and PE (17:0/17:0) for negative mode) and vortexed for 10 s. Next, 750 µL MTBE was added, vortexed again, and incubated at 4 °C for 10 min. After adding 188 µL ultrapure water, samples were vortexed for 20 s, centrifuged at 18,000 rpm (4 °C, 2 min), and the upper layer (350 µL) was transferred to a fresh tube. Finally, 120 µL methanol:toluene (9:1) was added, followed by vortexing (10 min), sonication (10 min), and centrifugation (18,000 rpm, 10 min). Prepared samples were analyzed with LC-MS.

### 2.5 Chromatographic and mass spectrometric conditions

LC analyses utilized an ACQUITY CSH C18 column (2.1 mm × 100 mm, 1.7 μm) at a 0.6 mL/min flow rate. Mobile phases for positive mode were (A) acetonitrile-water (6:4) + 10 mmol/L ammonium formate + 0.1% formic acid and (B) isopropanol-acetonitrile (9:1) + 10 mmol/L ammonium formate + 0.1% formic acid. Negative mode utilized (A) acetonitrile-water (6:4) + 10 mmol/L ammonium acetate and (B) isopropanol-acetonitrile (9:1) + 10 mmol/L ammonium acetate. For gradient elution, the following conditions were used: 0–2 min, 15%–30% B; 2–2.5 min, 30%–48% B; 2.5–11 min, 48%–82% B; 11–11.5 min, 82%–99% B; 11.5–12 min, 99% B; 12–13 min, 99%–15% B; and 13–15 min, 15% B. Column temperature was 65 °C, and injection volume was 5 µL (Peng et al., 2023). MS/MS spectra were acquired on a Q-Exactive Orbitrap mass spectrometer equipped with a heated electrospray ionization (HESI) source. Data were collected in dd-SIM (data-dependent SIM) mode. The acquisition parameters were as follows: resolution, 17,500 (at m/z 200); AGC target, 1 × 10^5^; maximum injection time, 50 ms; loop count, 4; isolation window, 1.0 m/z; fixed first mass, 60.0 m/z; and stepped normalized collision energies of 20, 30, and 40 eV. Spectral data were acquired in centroid mode.

### 2.6 Mass spectrometry conditions

Mass spectrometry employed a Q-Exactive Orbitrap-MS with HESI, operating in positive (+) and negative (−) ion modes. Key parameters were spray voltage (3.5 kV [+], 3.0 kV [−]), ion source temperature (306 °C [+], 325 °C [−]), capillary temperature (300 °C), nitrogen sheath (275 kPa) and auxiliary gases (104 kPa), S-lens RF level (50), and mass range (215–1,800 m/z) (Yang et al., 2019).

### 2.7 Data processing and lipid identification

The raw files in both positive and negative ion modes were converted to Abf format. Data processing was performed using MS-DIAL (v. 4.90) (Tsugawa et al., 2020). Parameters: start retention time: 1.5 min, end retention time: 27.5 min, mass range start: 215 Da, mass range end: 1,800 Da, MS1 centroid tolerance: 0.01 Da, smoothing level: 3 scans, minimum peak height: 10,000 amplitude, mass slice width: 0.1 Da, exact mass tolerance (MS1): 0.01 Da, exact mass tolerance (MS2): 0.05 Da, retention time alignment tolerance: 0.1 min, MS1 alignment tolerance: 0.025 Da (Yang et al., 2019).

For lipid identification, accurate mass and MS/MS matching were accomplished using the public LipidBlast library, including over 200,000 MS/MS spectra, covering 25 lipid sub-classes and several molecular species: BA (bile acids), BMP (bis(monoacylglycerol)phosphate), CAR (acylcarnitine), CE (cholesteryl esters), Cer (ceramides), CL (cardiolipins), DG (diacylglycerophosphates), FA (fatty acids), HexCer (hexosylceramides), LPC (lysophosphatidylcholine), LPE (lysophosphatidylethanolamine), LPG (lysophosphatidylglycerol), LPI (lysophosphatidylinositol), MG (monoacylglycerol), NAE (N-acylethanolamines), PC (phosphatidylcholine), PE (phosphatidylethanolamine), PG (phosphatidylglycerol), PI (phosphatidylinositol), SE (sulfate esters), SM (sphingomyelins), SPB (sphingoid base), ST (sterol esters), TG (triglycerides).

### 2.8 Normalization and statistical analysis

Multivariate analysis was performed using MetaboAnalyst 6.0 (https://www.metaboanalyst.ca) (Pang et al., 2021). After log transformation (base 10) and Pareto scaling, a statistical model was created utilizing TIC-normalized data (median). Differential lipids were selected based on a fold change > 2.0 or < 0.5 and P < 0.05. Enrichment analysis of differential lipids was performed using ChemRICH (http://chemrich.fiehnlab.ucdavis.edu/). Heatmaps and volcano plots were generated to depict the selected differential lipids. Statistical analysis was done using Graphpad Prism 8.0.2 software, with box plots illustrating the median, lower and upper quartiles, and the minimum and maximum values. Data from two unpaired groups were analyzed using the Mann-Whitney nonparametric test, and multiple comparisons were performed using one-way ANOVA.

## 3 Results

### 3.1 Analysis of lipid composition in blood

**FIGURE 2:**
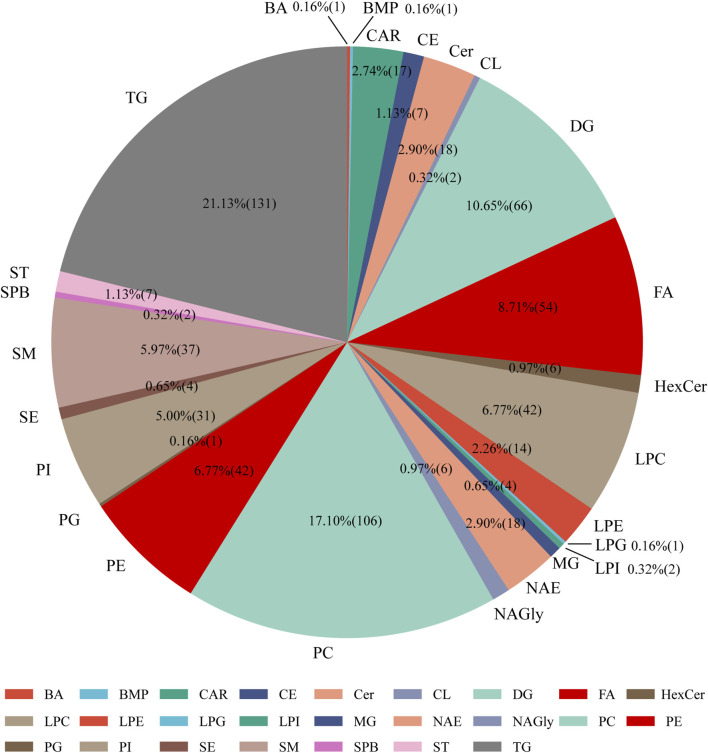
Distribution of lipid subclasses identified in serum samples from mice administered dexamethasone or saline solution (subcutaneous injection) for 4 days. Abbreviations: BA: bile acids, BMP: bis(monoacylglycerol)phosphate, Car: acylcarnitine, CE: cholesteryl esters, Cer: ceramides, CL: cardiolipins, DG: diacylglycerophosphates, FA: fatty acids, HexCer: hexosylceramides, LPC: lysophosphatidylcholine, LPE: lysophosphatidyl-ethanolamine, LPG: lysophosphatidylglycerol, LPI: lysophosphatidylinositol, MG: monoacylglycerol, NAE: N-acylethanolamines, NAGly: N-arachidonoylglycine, PC: phosphatidylcholine, PE: phosphatidylethanolamine, PG: phosphatidylglycerol, PI: phosphatidylinositol, SE: sulfate esters, SM: sphingomyelins, SPB: sphingoid base, ST: sterol esters, TG: triglycerides.

We implemented MS-DIAL software along with the LipidBlast library to detect lipid species from LC-MS analyses conducted in both positive and negative ion modes. The identified lipid classes included phosphatidylcholine (PC), phosphatidylethanolamine (PE), phosphatidylinositol (PI), phosphatidylglycerol (PG), lysophosphatidylcholine (LPC), lysophosphatidylethanolamine (LPE), and lysophosphatidylglycerol (LPG). In serum samples, we detected 439 lipid species in positive ion mode and 221 lipid species in negative ion mode, totaling 626 lipid species. Among them, glycerophospholipids contributed roughly 38%, glycerolipids accounted for 20.9%, sphingolipids constituted 10%, fatty acids made up 19.8%, sterols were 3%, and glycolipids represented 3.6% of the total identified lipids (Figure 2).

### 3.2 UHPLC-Q-Exactive Orbitrap MS method validation

To verify data dependability and assess instrument stability, we performed three quality control processes. First, a dual-needle quality control (QC) sample balancing equipment was used during sample preparation prior to the injection of experimental samples. Second, we continually monitored the peak intensities of internal standards across all samples and computed the relative standard deviation (RSD). Third, blank solvent and QC samples were inserted after every 15 experimental samples to evaluate any carryover and system drift.

**FIGURE 3:**
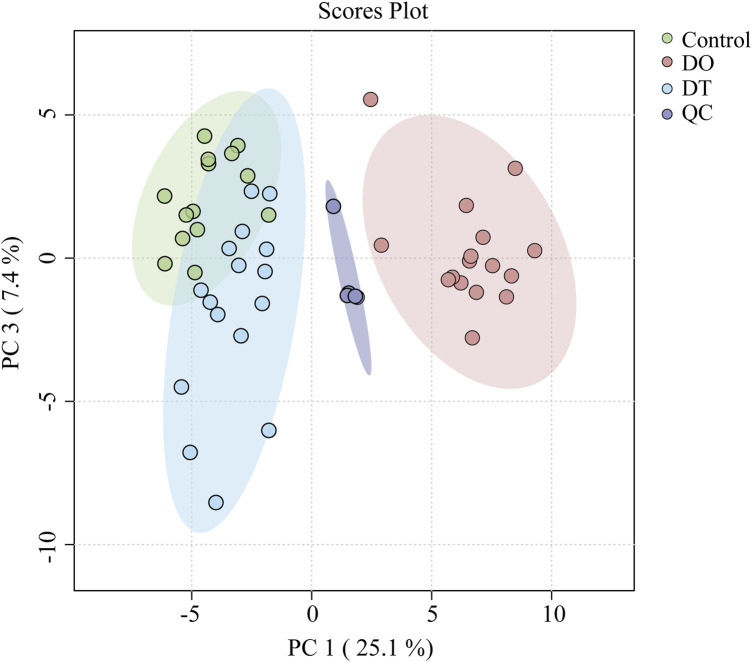
Principal component analysis (PCA) score plot of serum lipidomics data in positive and negative ion modes. Each point represents an individual sample: Control (green), DO (red), DT (blue), and QC (purple). Ellipses indicate the 95% confidence regions for each group.

Principal component analysis (PCA) was performed on the QC data matrix to evaluate system stability and reproducibility. The PCA score graphs demonstrated tight clustering of QC samples in both positive and negative ion modes, suggesting high analytical consistency and instrument stability throughout the experimental run (Figure 3). Additionally, the RSD values for the internal standards lysoPE (17:1) (batch number LM171LPE-11) and PE (17:0/17:0) were below 10% in both ionization modes, further demonstrating the endurance of the UHPLC-Q-Exactive Orbitrap-MS technology.

### 3.3 Untargeted lipidomics metabolic analysis

We conducted an untargeted lipidomic analysis using UPLC-MS to assess the influence of dexamethasone on serum lipid profiles. Initially, an unbiased principal component analysis (PCA) was performed to reveal the total lipidomic variances. As indicated in Figure 3, considerable separation was found across the DO, DT, and control groups, revealing significant metabolic changes in blood lipid composition among these groups. In positive ion mode, after a 7-day washout period, the changes of lipids intend to recover. These results indicated dexamethasone intervention significantly induced changes in serum lipids in mice, and after a 7-day washout period, changes of lipids in the serum were still existed compared with those in the control groups.

**FIGURE 4:**
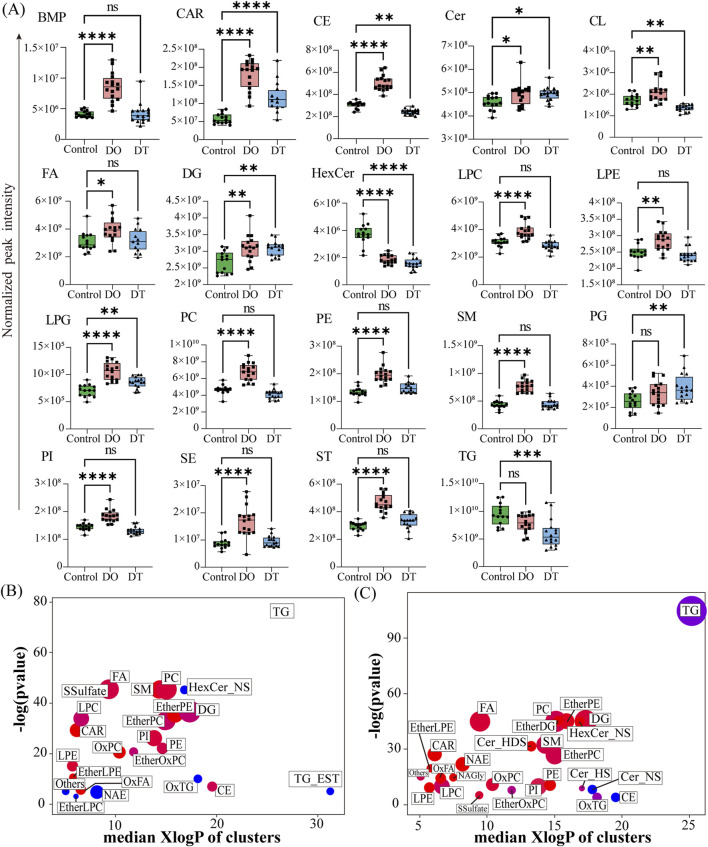
Summary of Differentially Expressed Lipids in Serum. **(A)** Box plots showing the normalized peak intensities of lipid classes in serum. Each box plot represents the distribution of the entire lipid class, rather than individual lipids. The boxes span from the 25th to the 75th percentile, with the line inside the box indicating the median value. Statistical significance between groups was assessed using the Mann–Whitney U test; p < 0.05. **(B)** Enrichment analysis of differentially expressed lipid classes between the DO and Control groups. **(C)** Enrichment analysis of differentially expressed lipid classes between the DT and Control groups. Enrichment p-values were calculated using the Kolmogorov–Smirnov test. Each node represents a lipid cluster; node size corresponds to the number of lipids within the cluster. Node colors indicate the regulation direction: red for upregulated, blue for downregulated, and purple for clusters containing both up- and downregulated lipids. Abbreviations: BMP: bis(monoacylglycerol)phosphate, Car: acylcarnitine, CE: cholesteryl esters, Cer: ceramides, Cer_HDS: ceramide, hydroxylated dihydrosphingosine, Cer_HS: ceramide, hydroxylated sphingosine, Cer_NS: ceramide, non-hydroxy fatty acid-sphingosine, CL: cardiolipins, DG: diacylglycerophosphates, EtherDG: ether-linked diacylglycerol, EtherLPC: ether-linked lysophosphatidylcholine, EtherLPE: ether-linked lysophosphatidylethanolamine, EtherOxPC: oxidized ether-linked phosphatidylcholine, EtherPC: ether-linked phosphatidylcholine, EtherPE: ether-linked phosphatidylethanolamine, FA: fatty acids, HexCer: hexosylceramides, HexCer_NS: hexosylceramide (non-hydroxy fatty acid-sphingosine), LPC: lysophosphatidylcholine, LPE: lysophosphatidylethanolamine, LPG: lysophosphatidylglycerol, NAE: N-acylethanolamines, NAGly: N-arachidonoylglycine, OxFA: oxidized fatty acid, OxPC: oxidized phosphatidylcholine, OxTG: oxidized triglyceride, PC: phosphatidylcholine, PE: phosphatidylethanolamine, PG: phosphatidylglycerol, PI: phosphatidylinositol, SE: sulfate esters, SM: sphingomyelins, SSulfate: sulfatides, ST: sterol esters, TG: triglycerides, TG_EST: triglyceride ester derivatives.

Out of 25 lipid subtypes found in serum, 19 indicated considerable alterations. As shown in Figure 4A, following 4 days of dexamethasone administration, 16 kinds of lipids in the serum showed significantly increased compared with those in the control groups, Among these lipids, 10 lipids were returned to the normal level, including BMP, FA, LPC, LPE, PC, PE, SM, PI, SE and ST after a 7-day washout period; and 4 lipids were still increased, including Cer, DG, LPG, and PG after a 7-day washout period. Moreover, levels of CE and CL were decreased after a 7-day washout period (Figure 4A).

Following 4 days of dexamethasone administration, levels of HexCer and TG in the serum showed decreased compared with those in controls, and both these two lipids were still keeping decreased levels after a 7-day washout period, indicating that these lipids might also be related to the side effects of dexamethasone (Figure 4A).

To further evaluate the biological ramifications of these lipid modifications, enrichment analysis was done on differentially expressed lipids meeting the criterion of fold change (FC) > 2.0 or ≤ 0.5 and p < 0.05. As indicated in Figures 4B,C, each node indicates a lipid group with significant modifications, with node size denoting the number of lipid species involved. This investigation demonstrated considerable modifications in phospholipids, sphingolipids, glycolipids, fatty acids, glycerophospholipids, and glycerolipids following 4 days of dexamethasone administration. Glycerolipids, notably TG, were among the most changed lipid classes, suggesting dexamethasone’s strong influence on this category.

After a 7-day washout period, LPC, NAE, and HexCer still indicated a downregulation tendency, while FA, PC, SM, PE, CAR, and LPE were still raised. Additionally, both overexpression and downregulation patterns were identified in LPC, DG, and TG subclasses. In the DT group, 27 lipid categories revealed notable changes compared to the Control, with 21 categories upregulated and 3 downregulated (Figure 4C).

These statistics indicate the vast and dynamic impact of dexamethasone on serum lipid metabolism, both during and after treatment exposure.

### 3.4 Heatmap and volcano plot analyses of lipid alterations

**FIGURE 5:**
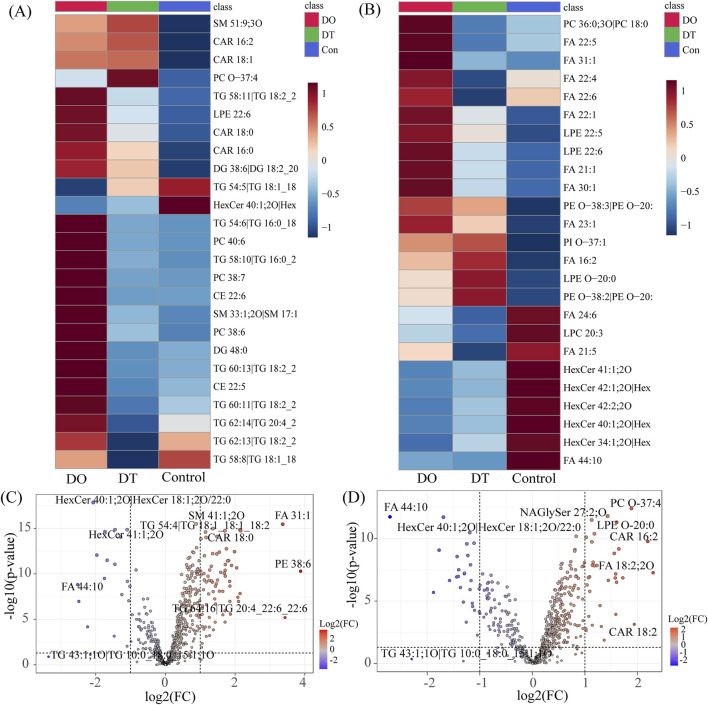
Effects of Dexamethasone on Serum Lipid Profiles. **(A)** Heatmap of differentially expressed lipids in serum under positive ion mode. **(B)** Heatmap of differentially expressed lipids in serum under negative ion mode. Each square represents the normalized intensity of a specific phospholipid in individual samples. Red indicates increased abundance, while blue indicates decreased abundance. **(C)** Volcano plot of differentially expressed lipids between the DO and Control groups. **(D)** Volcano plot of differentially expressed lipids between the DT and Control groups. Each dot represents a lipid species, with the x-axis indicating log_2_ fold change (FC) and the y-axis indicating −log_10_ p-value. Red dots represent significantly upregulated lipids (FC > 1.5, p < 0.05); blue dots represent significantly downregulated lipids (FC < 0.67, p < 0.05). Abbreviations: CAR: acylcarnitine, CE: cholesteryl esters, DG: diacylglycerophosphates, FA: fatty acids, HexCer: hexosylceramides, LPE: lysophosphatidylethanolamine, NAGlySer: N-arachidonoyl glycine serine, PC: phosphatidylcholine, PE: phosphatidylethanolamine, PI: phosphatidylinositol, SM: sphingomyelins, TG: triglycerides.

Heatmap analysis demonstrated diverse lipidomic patterns across the three mouse groups (Figures 5A,B). Each square in the heatmap shows the normalized intensity of a certain lipid species in independent samples. The color gradient from red to blue shows a decline in lipid intensity, with deeper red denoting more abundance and blue signaling lesser abundance. Under the threshold of *p* < 0.05, the positive ion mode heatmap (Figure 5A) showed that 21 lipids were upregulated after 4 days of dexamethasone administration. Among these increased lipids, 18 lipids intend to recover after a 7-day washout period, and 3 lipids were still increased including SM 51:9, CAR 16:2, and CAR 18:1. Three lipids were decreased compared with those in the control, including TG 54:5, TG 58:8 and HexCer 40:1, and all these lipids were still keeping decreased after a 7-day washout period.

In the negative ion mode (Figure 5B), 16 lipids were upregulated and 9 lipids were downregulated (FDR < 0.05) after 4 days of dexamethasone administration. Among these increased lipids, 9 lipids intend to recover after a 7-day washout period, and 7 lipids were still increased. The decreased 9 lipids compared with those in the control, and all these lipids were still keeping decreased after a 7-day washout period.

Volcano plot analysis (Figures 5C,D) indicated the distribution of differentially expressed lipids based on log_2_ fold change and statistical significance. Red dots depict dramatically increased lipids, while blue dots show downregulated lipids in the dexamethasone-exposed groups. After 4 days 4 days of dexamethasone administration, 73 lipid species satisfied the significance criterion (fold change > 2.0 or < 0.5, p < 0.05). The most significantly downregulated lipid was FA 44:10 (fold change = 0.175), while the most significantly increased lipid was PE 38:6 (fold change = 14.728). After a 7-day washout period, 47 lipid species surpassed the barrier, with FA 44:10 again exhibiting the largest decline (fold change = 0.15321) and FA 18:2; 2O indicating the most noteworthy elevation (fold change = 4.6615).

## 4 Discussion

In this study, we demonstrate that dexamethasone treatment drastically changes the serum lipidome. Dexamethasone is a potent anti-inflammatory medication whose broad clinical use has caused considerable worry regarding its metabolic adverse effects (Pofi et al., 2023). Our lipidomic research reveals that even short-term, clinically equivalent dosage of dexamethasone can impact lipid homeostasis at a systems level. We revealed that over 80% of evaluated lipid subclasses were significantly changed after dexamethasone administration, underlining the widespread influence of this glucocorticoid on lipid metabolism. Importantly, this is the first indication of systemic lipid changes in serum induced by dexamethasone, expanding prior investigations of glucocorticoid effects on isolated tissues and parameters.

Key lipid abnormalities were detected at both early (4 days) and later (11 days) time points following dexamethasone treatment. After 4 days of therapy, many lipid classes–including BMP, CAR, HexCer, LPG, PC, PE, SM, PI, SE, and ST–showed considerable alterations relative to controls. After a 7-day washout period (equivalent to∼28 drug half-lives), many of these disturbances persisted or evolved: CE, CL, HexCer, and TG were significantly decreased in the dexamethasone-treated group, whereas CAR, Cer, DG, LPG, and PG remained elevated compared to controls. These patterns represent a dynamic remodeling of the lipidome, where some lipid abnormalities resolve when the steroid is removed (e.g., CE and TG levels), while others reveal longer-lasting metabolic modifications generated by dexamethasone.

We then explored the biological repercussions of these lipidomic changes. Several altered lipid classes are recognized to play key roles in metabolism and sickness, offering suggestions about how dexamethasone’s lipid modifications can translate into adverse outcomes. We noticed a substantial decrease in serum CE following the dexamethasone treatment period. CE are the esterified storage form of cholesterol, carried in lipoproteins (especially LDL and HDL) and transferred by cholesteryl ester transfer protein (CETP) to maintain cholesterol balance. A decline in circulating CE can reflect dexamethasone-driven redistribution of cholesterol–for instance, increased absorption by tissues or less esterification in the liver. While on the surface a lower CE level could seem positive, it must be interpreted in context. Cholesterol homeostasis is highly regulated, and deviations can have atherogenic implications. Excess buildup of CE in arterial walls leads to foam cell formation, the characteristic of early atherosclerotic plaques (Prete and Bancos, 2021). Conversely, a reduction in plasma CE as seen here can reflect rapid clearance of cholesterol into tissues or modified lipoprotein metabolism. Dexamethasone is known to upregulate hepatic LDL receptors transiently, which could boost LDL-C uptake and lower circulating CE. However, extended glucocorticoid exposure often elevates atherogenic lipids (Sholter and Armstrong, 2000). It is conceivable that our 7-day post-dexamethasone time point reflects a healing period where lipids like CE and TG were brought down due to acute metabolic changes. The total effect of dexamethasone on cholesterol might therefore be biphasic - an early reduction followed by eventual rise with chronic medication, as reported clinically (Sholter and Armstrong, 2000). Regardless, the modification of CE metabolism by dexamethasone shows that even short-term treatment might impede cholesterol transport. Given that increased CE in LDL induces atherosclerosis by depositing cholesterol in macrophages (Ghosh et al., 2010), the alterations we discover could influence long-term cardiovascular risk if they stay or if compensatory hyperlipidemia occurs after steroid withdrawal.

HexCer are sphingolipids involved in cell membrane structure and signaling. Dexamethasone’s influence on HexCer was complex: levels were initially boosted at 4 days then substantially lower than control after 7 days. Sphingolipid metabolism is critically relevant to metabolic health. Glycosphingolipids such HexCer and gangliosides have been established as contributing to obesity-induced insulin resistance (Langeveld and Aerts, 2009). Excess HexCer can impair insulin receptor signaling and promote pro-inflammatory pathways. For instance, animal studies suggest that rising HexCer content in tissues is connected with insulin resistance and glucose intolerance. Consistently, pharmacological reduction of glycosphingolipid synthesis promotes insulin sensitivity in obese rodents (Langeveld and Aerts, 2009). In this work, transitory elevation of HexCer during dexamethasone therapy may represent stress-induced sphingolipid turnover, potentially contributing to insulin resistance. By day 7, HexCer levels dropped, suggesting possible negative feedback or increased catabolism. Given that several HexCer species are markers of metabolic syndrome and type 2 diabetes, and are associated to inflammation and endothelial dysfunction, these differences imply that dexamethasone modifies sphingolipid balance. Such changes may impair metabolic balance and immune regulation.

Dexamethasone dramatically raised levels of LPG, a minor lysophospholipid implicated in immunological control. LPG was greatly elevated at both 4 and 7 days post-treatment, indicating a sustained metabolic effect. As a result of phosphatidylglycerol hydrolysis, LPG can serve as a signaling molecule. These findings show that dexamethasone increases phospholipid turnover, resulting to the development of bioactive lysophospholipids like LPG, which may modify immune responses through interactions with surface receptors such as Toll-like receptor 2 (TLR2) (Makide et al., 2014). While specific receptors for LPG are poorly described, research have indicated that LPG itself can impact leukocyte function. In human phagocytes (neutrophils and monocytes), LPG was demonstrated to inhibit chemoattractant-induced migration and inflammatory cytokine (IL-1β) production (Shim et al., 2009), hence lowering certain inflammatory responses. Conversely, in natural killer cells, LPG serves as a chemoattractant that increases migration via G-protein coupled receptors (Jo et al., 2008). These observations imply that LPG works as a bioactive lipid involved in immune cell trafficking and activation. The sustained elevation of LPG following dexamethasone treatment may contribute to its complicated immunomodulatory effects. While increasing LPG could lower certain innate inflammatory responses—consistent with dexamethasone’s anti-inflammatory role—it might also promote aberrant immune cell recruitment or chronic low-grade inflammation. Elevated LPG has been connected to autoimmune and viral illnesses, however *in vivo* evidence remains rare. Our results indicate that dexamethasone generates lysophospholipid imbalance, potentially compromising immune homeostasis. Persistent LPG buildup may lead to minor immune dysregulation long after medication finishes, contributing to dangers such as steroid withdrawal syndrome or heightened infection susceptibility. Moreover, persistent lysophospholipid-driven inflammation could worsen atherogenesis and metabolic dysfunction, underlining the significance of monitoring and regulating these lipid alterations during glucocorticoid therapy.

Collectively, these lipid-specific discoveries indicate how dexamethasone’s systemic effects on lipids might translate into clinical repercussions. The observed increase in atherogenic lipids (e.g., ceramides) and pro-inflammatory mediators (e.g., lysophosphatidylglycerol) provides mechanistic support for the well-documented side effect profile of glucocorticoids, including hyperlipidemia, insulin resistance, and elevated cardiovascular risk (Sholter and Armstrong, 2000). Likewise, the alteration of cholesterol and sphingolipid metabolism may underlie subtler effects on cell membrane composition and immune cell activity that lead to immunosuppression and tissue dysfunction after steroid therapy. From a translational approach, our data offer possible biomarkers and therapeutic targets. For instance, monitoring serum HexCer during glucocorticoid treatment could serve as an early signal of metabolic derangements, enabling preventive interventions (such as dietary modifications or insulin-sensitizing medications). Similarly, the persistent rise of LPG suggests that adjunct therapy targeting at regulating lysophospholipid levels (e.g., modulators of lipid hydrolases or receptors) can attenuate certain negative effects. The lipidomic changes mapped here also propose routes for further research into glucocorticoid biology—for example, investigating whether genetic variations in lipid metabolism influence individual susceptibility to steroid-induced diabetes or dyslipidemia.

In conclusion, utilizing a cutting-edge lipidomics method, we provide the first complete proof that dexamethasone intervention generates extensive modification of the serum lipidome. Even short-term exposure to a therapeutically adequate amount resulted in substantial modifications in glycerolipids, fatty acyls, sphingolipids, and sterols. These alterations offer a molecular explanation for dexamethasone’s metabolic side effects and widen our understanding of its pharmacological action beyond gene regulation and protein creation, into the world of lipid signaling and metabolism. Our findings thus not only highlighted the necessity of meticulous metabolic monitoring during glucocorticoid therapy but also creates a platform for developing ways to extract the positive anti-inflammatory benefits of dexamethasone from its deleterious metabolic consequences (Pofi et al., 2023). By understanding the specific lipid pathways affected by dexamethasone, our work adds to more informed usage of glucocorticoids and the design of safer anti-inflammatory drugs in the future.

## Data Availability

The data presented in the study are deposited in the the Figshare repository, doi: https://doi.org/10.6084/m9.figshare.29972671

## Glossary

BA: bile acids
BMP: bis(monoacylglycerol)phosphate
Car: acylcarnitine
CE: cholesteryl esters
Cer: ceramides
Cer_HDS: ceramide, hydroxylated dihydrosphingosine
Cer_HS: ceramide, hydroxylated sphingosine
Cer_NS: ceramide, non-hydroxy fatty acid-sphingosine
CETP: cholesteryl ester transfer protein
CL: cardiolipins
dd-SIM: data-dependent SIM
DG: diacylglycerophosphates
EtherDG: ether-linked diacylglycerol
EtherLPC: ether-linked lysophosphatidylcholine
EtherLPE: ether-linked lysophosphatidylethanolamine
EtherOxPC: oxidized ether-linked phosphatidylcholine
EtherPC: ether-linked phosphatidylcholine
EtherPE: ether-linked phosphatidylethanolamine
FA: fatty acids
FC: fold change
HDL: high-density lipoprotein
HESI: heated electrospray ionization
HexCer: hexosylceramides
HexCer_NS: hexosylceramide (non-hydroxy fatty acid-sphingosine)
LDL: low-density lipoprotein
LPC: lysophosphatidylcholine
LPE: lysophosphatidylethanolamine
LPG: lysophosphatidylglycerol
LPI: lysophosphatidylinositol
MG: monoacylglycerol
NAE: N-acylethanolamines
NAGly: N-arachidonoylglycine
OxFA: oxidized fatty acid
OxPC: oxidized phosphatidylcholine
OxTG: oxidized triglyceride
PC: phosphatidylcholine
PCA: principal component analysis
PE: phosphatidylethanolamine
PG: phosphatidylglycerol
PI: phosphatidylinositol
QC: quality control
RSD: relative standard deviation
SE: sulfate esters
SM: sphingomyelins
SPB: sphingoid base
SSulfate: sulfatides
ST: sterol esters
TG: triglycerides
TG_EST: triglyceride ester derivatives
TLR2: Toll-like receptor 2
UHPLC-Q-Exactive Orbitrap-MS: ultra-high-performance liquid chromatography connected to quadrupole-Orbitrap mass spectrometry
